# HIGH RISK OF NEW PSYCHIATRIC DISORDERS AND SUICIDAL BEHAVIOR IN DEMENTIA

**DOI:** 10.1093/geroni/igz038.1150

**Published:** 2019-11-08

**Authors:** Amy Lai, Yixia Li, Amy Byers

**Affiliations:** SF Veterans Affairs Health Care System, San Francisco, California, United States

## Abstract

Occurrence of new mental health (MH) disorders in patients with dementia is neglected, with next to nothing known. We examined association between dementia diagnosis and risk of new psychiatric disorders and suicide, and MH services use. We merged four national databases from US Department of Veterans Affairs. Sample included 2,529,181 patients (≥50 years) in fiscal years (FY) 2012-2013 with no MH disorders. Dementia, psychiatric disorders (mood, anxiety, substance), suicidal behavior (ideation, plan, attempt, death by suicide) were identified by ICD-9/10 codes and national suicide databases. Hazard ratios (HR) were estimated using Cox proportional hazard models, with time-to-event defined as age at first diagnosis of MH disorder during FY 2014-2016. Analyses adjusted for medical/sociodemographic factors. Compared to those without dementia, dementia patients showed roughly 2-fold increased risk of new mood (HR: 2.19, 95% Wald CI: 2.15-2.24, p<.001) or anxiety (HR: 1.56, 95% CI: 1.50-1.63, p<.001) disorders. Recent dementia diagnosis was associated with highest risk of these disorders than prior or no diagnosis; for example, patients with recent diagnosis showed 72% greater risk of anxiety disorders (HR: 1.72, 95% CI: 1.63-1.81, p<.001). Although patients with prior dementia diagnosis had lower risk of suicidal behavior, risk increased with recent dementia diagnosis. However, dementia patients with new MH disorders showed little MH services use (< 20%). Patients with dementia have increased risk of new MH disorders, especially recent dementia diagnosis. Furthermore, MH services are underutilized, highlighting critical need for integration of such services in caring for dementia patients.

